# Type 2 Diabetes Mellitus and Cardiometabolic Prospects: A Rapid Narrative Review

**DOI:** 10.7759/cureus.65808

**Published:** 2024-07-30

**Authors:** Kona Chowdhury, Susmita Sinha, Rahnuma Ahmad, Halyna Lugova, Miral Mehta, Santosh Kumar, Mainul Haque

**Affiliations:** 1 Department of Pediatrics, Enam Medical College Hospital, Dhaka, BGD; 2 Department of Physiology, Enam Medical College Hospital, Dhaka, BGD; 3 Department of Physiology, Medical College for Women and Hospital, Dhaka, BGD; 4 Department of Medicine and Health Sciences, UCSI (University College Sedaya International) University Bandar Springhill Campus, Port Dickson, MYS; 5 Department of Pedodontics and Preventive Dentistry, Karnavati School of Dentistry, Karnavati University, Gandhinagar, IND; 6 Department of Periodontology and Implantology, Karnavati School of Dentistry, Karnavati University, Gandhinagar, IND; 7 Department of Research, Karnavati Scientific Research Center (KSRC) School of Dentistry, Karnavati University, Gandhinagar, IND; 8 Department of Pharmacology and Therapeutics, National Defence University of Malaysia, Kuala Lumpur, MYS

**Keywords:** psychological and behavioral interventions, weight reduction, physical activity, healthy diet, lifestyle modifications, dyslipidemia, insulin resistance, cardiovascular diseases, type 2 diabetes mellitus, cardiometabolic syndrome

## Abstract

Cardiometabolic syndrome (CMS), type 2 diabetes mellitus (T2DM), and cardiovascular diseases are among the major altruists to the international liability of disease. The lifestyle and dietary changes attributable to economic growth have resulted in an epidemiological transition towards non-communicable diseases (NCDs) as the leading causes of death. Low- and middle-income countries (LMICs) bear a more substantial disease burden due to limited healthcare sector capacities to address the rapidly growing number of chronic disease patients. The purpose of this narrative review paper was to explore the interrelationships between CMS, T2DM, and cardiovascular impairments in the context of NCDs, as well as major preventative and control interventions. The role of insulin resistance, hyperglycemia, and dyslipidemia in the pathogenesis of T2DM and the development of severe cardiovascular impairments was highlighted. This paper elaborated on the pivotal role of lifestyle modifications, such as healthy diets and physical activity, as cornerstones of addressing the epidemics of metabolic diseases. Foods high in calories, refined sugar, red meat, and processed and ready-to-eat meals were associated with an amplified risk of CMS and T2DM. In contrast, diets based on fruits, legumes, vegetables, and whole grain, home-cooked foods demonstrated protective effects against metabolic diseases. Additionally, the role of a psychological and behavioral approach in addressing metabolic diseases was highlighted, especially regarding its impact on patient empowerment and the patient-centered approach to preventative and therapeutic interventions.

## Introduction and background

In recent decades, there has been an upsurge in the number of non-communicable diseases (NCDs). Globally, NCDs are recognized as the leading public health challenge and the main cause of death across various populations. The top-ranking NCDs contributing to the global disease burden include cardiovascular diseases (CVDs), chronic respiratory diseases, diabetes mellitus (DM), and cancers. Several recent studies have reported a growing trend of premature mortality attributable to NCDs in certain countries [1-5]. The most rapid growth has been observed in low- and middle-income countries (LMICs) due to the lack of capabilities of the health systems to cope with the increasing number of patients with chronic diseases [6,7]. The rise in NCDs in these countries is associated not only with aging populations but also with the detrimental effects of uncontrolled urbanization, aggressive marketing, and unfair trade, which result in unhealthy dietary patterns, excessive consumption of tobacco and alcohol, and increasingly sedentary lifestyles [8].

**Figure 1:**
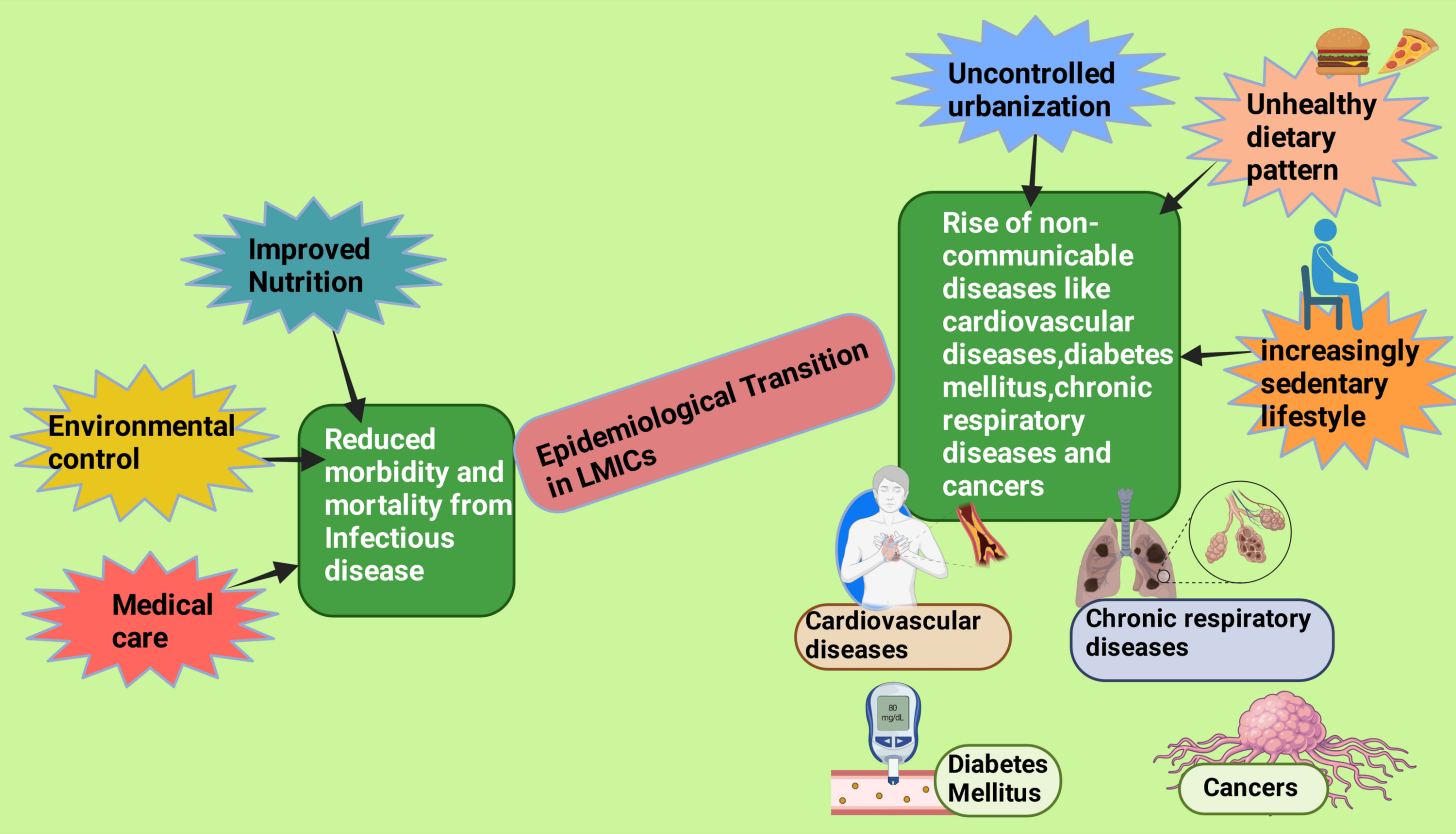
Diagram depicting the factors associated with the epidemiological transition in low-income countries with the reduction in infectious disease morbidity and mortality and the development of non-communicable diseases Note: This figure was drawn using the premium version of BioRender [11] (https://biorender.com, accessed on July 18, 2024) with the agreement license number DF272Q22H9. Image credit: Rahnuma Ahmad LMICs: Low- to middle-income countries

One of the challenges arising from economic development is how it is reflected in public health. On the one hand, the ultimate outcomes of a growing economy and technological advances are improved nutrition, environmental control, and medical care, resulting in reduced morbidity and mortality from infectious diseases, especially those potentially spreading epidemically and affecting significant proportions of the population. On the other hand, lifestyle changes brought about by economic development and globalization are considerable contributors to an increase in NCDs [9]. The process of changing population patterns concerning the leading causes of death is known as the epidemiological transition. Currently, LMICs are entering the third stage of epidemiological transition (Figure 1), when chronic diseases take over as the leading causes of death, mainly attributable to the change in the environment in which populations live and the aging aftereffects of economic growth [10].

Cardiometabolic syndrome (CMS) is one of the chronic conditions that has grown in public health significance in the past decades. This is primarily attributed to its role as a predisposing factor in the pathogenesis of CVD and type 2 diabetes mellitus (T2DM), accounting for a large proportion of excess global mortality [12]. T2DM possesses shared pathology with CMS that predisposes individuals to diabetic cardiomyopathy and atherosclerotic CVDs. These pathological issues frequently promote heart failure through long-standing blood pressure overburden and coronary impediment. Pathogenetic setups are principally associated with “hyperglycemia and chronic sustained hyperinsulinemia” [13]. These pathological issues recurrently modify “intracellular signaling pathways, redox status, metabolic profiles, energy production, increased susceptibility to ischemia, and extracellular matrix remodeling” [13,14]. Additionally, a growing number of studies have shed light on the mechanisms of CMS development and the role of microbiota and inflammation, the correlation of CMS with other medical conditions, and novel therapeutical modalities [15,16]. This paper aims to explore the complex interplay between cardiovascular and T2DM complications of CMS in the context of NCDs and the lifestyle and dietary changes needed to control the epidemic spread of these medical conditions. Other risk factors that promote CMS include prolonged low-grade inflammation, hyperlipidemia, obesity, persistent high blood pressure requiring medication [13], poor physical activity, desk-bound jobs, and surfeit fattiness [17-19]. 

## Review

Materials and methods

**Figure 2:**
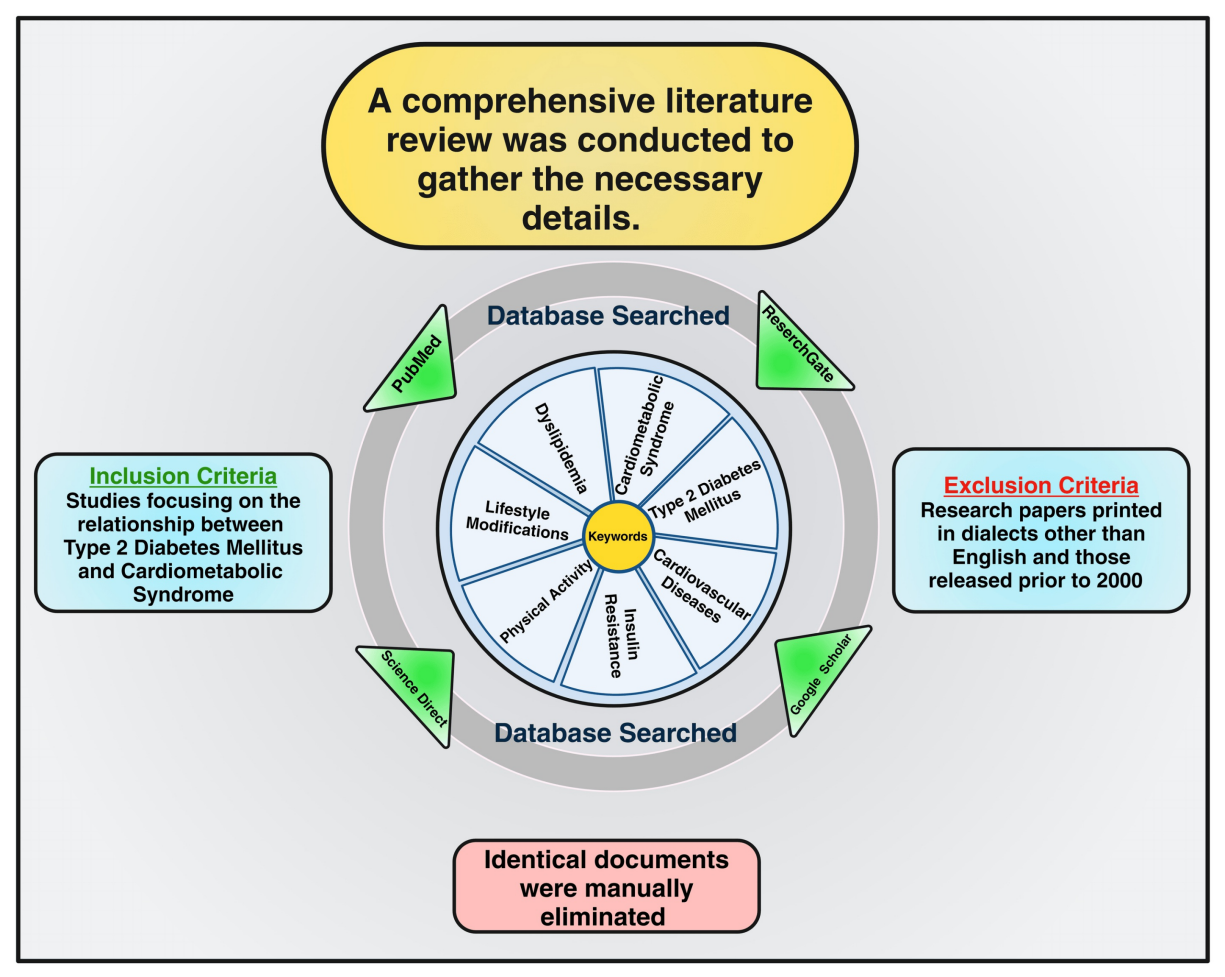
Chart showing the methodology of this paper Note: This figure has been drawn using the premium version of BioRender [11] (https://biorender.com, accessed on July 1, 2024), with the agreement license number UR2709IWJJ. Image credit: Susmita Sinha

This review paper studied the links among NCDs, important preventive and control strategies, and cardiovascular consequences in CMS and T2DM. Online databases were searched using PubMed, ResearchGate, Google Scholar, and Science Direct. The reference list of relevant articles was looked through to look up more literary works. "Cardiometabolic Syndrome," AND "Type 2 Diabetes Mellitus", AND "Cardiovascular Diseases," "Insulin Resistance," AND "Dyslipidemia," AND "Lifestyle Modifications," AND "Healthy Diet," AND "Physical Activity," AND "Weight Reduction," AND "Psychological and Behavioral Interventions" were among the search terms used. Dissertations published before 2000 and those issued in languages other than English were not accepted. The papers' appropriateness for inclusion in this study was thoroughly examined before submission. Following an independent examination and assimilation of the proposed literary works, a follow-up conversation was scheduled to address any questions, incorrect information, or biases about the specific pieces (Figure 2).

Review of literature

Global Health Implications of CMS

**Figure 3:**
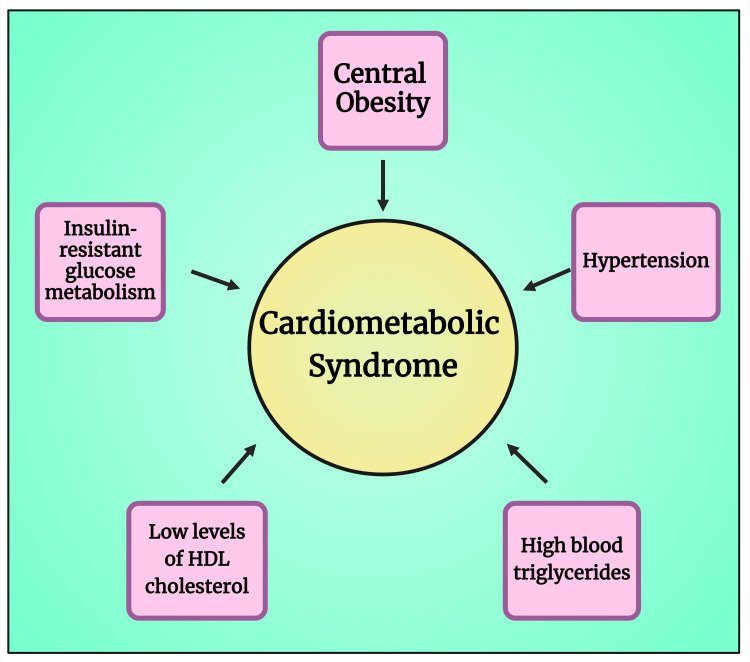
Diagram showing the health disorders related to cardiometabolic syndrome Note: This figure has been drawn using the premium version of BioRender [11] (https://biorender.com, accessed on March 28, 2024) with the agreement license number UI26MQRJB1. Image credit: Susmita Sinha

CMS includes a group of health disorders: central obesity, insulin‐resistant glucose metabolism, atherogenic dyslipidemia (high blood triglycerides and low levels of high-density lipoprotein cholesterol (HDL-C)), and hypertension (Figure 3) [13,20]. CMS is often called metabolic syndrome (MetS), syndrome X, or insulin resistance (IR) syndrome. This syndrome is strongly related to T2DM, atherosclerotic CVDs, and cardiomyopathy [13,21]. It is frequently reported that individuals with T2DM lead to grave situations of CMS [22,23]. Globally, DM is continuing to rise at full tilt and is emerging as a public health distress because of the high level of morbidity and mortality [24]. It has been reported that 80% of 463 million adult diabetic patients are from LMICs [25,26]. Additionally, in LMICs, limited access to healthcare, especially with respect to NCDs, including DM, increases both disease burden and death rates [27-29].

IR, Dyslipidemia, and Cardiovascular Complications of T2DM

**Figure 4:**
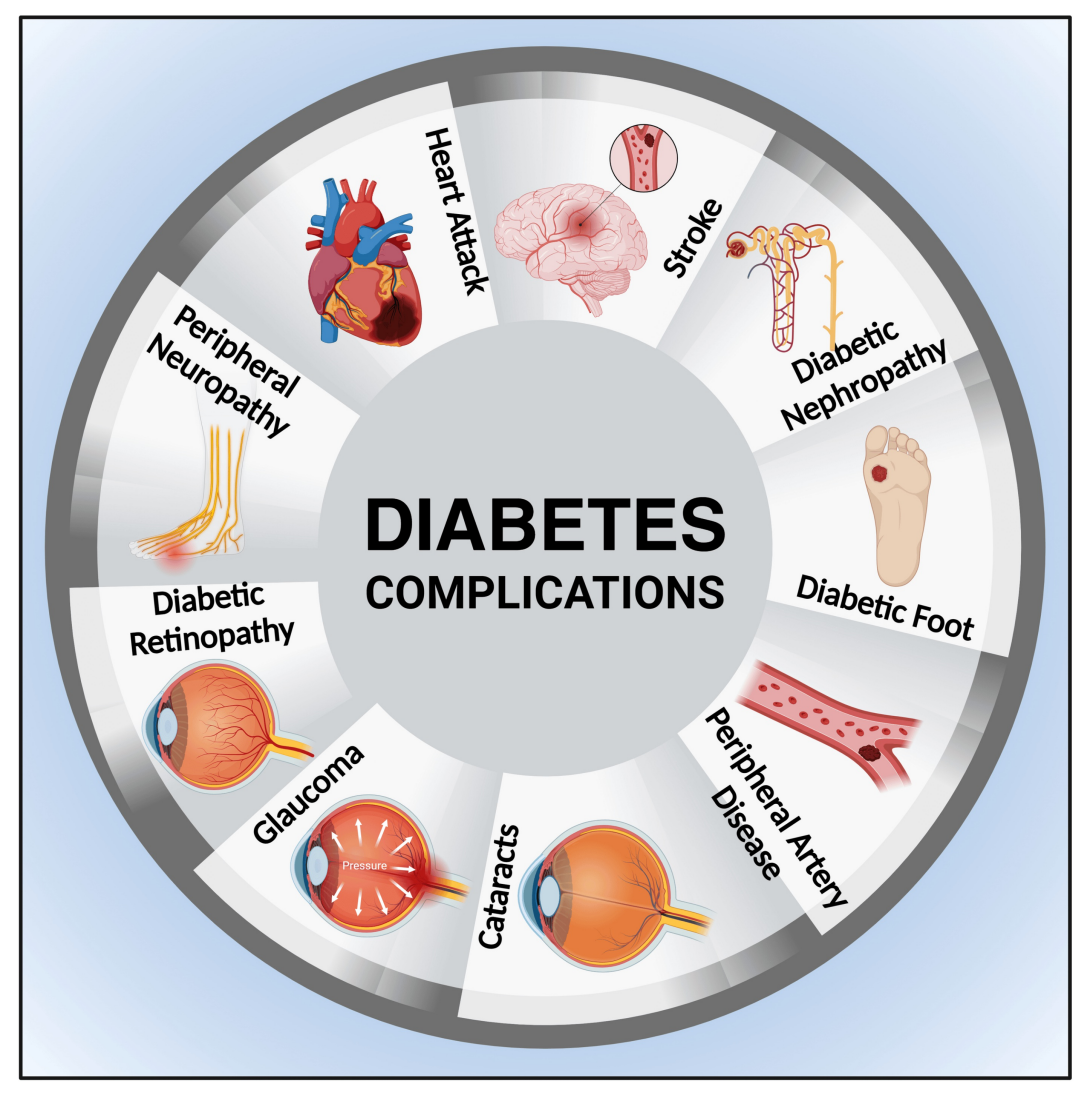
Illustration showing the complications of diabetes Note: This figure has been drawn using the premium version of BioRender [11] (https://biorender.com, accessed on March 29, 2024) with the agreement license number KS26MSYVE9. Image credit: Susmita Sinha

T2DM is drawing a connection between several metabolic aberrations [30,31] that comprise IR and fall-off insulin synthesis [18,32,33], leading to sustained high blood glucose levels and the breach of the normal lipid metabolism process (e.g., cholesterol, low-density lipoprotein cholesterol (LDL-C), triglycerides, and HDL-C) [34], which germinates hypertension, coronary artery disease (CAD), myocardial infarction (MI), stroke, peripheral arterial disease (PAD), and sequentially glares CVD [33,35]. It has been reported that individuals with an average glycemic index (much before the diagnosis of T2DM) frequently have dyslipidemia, e.g., low HDL-C, high LDL-C, and triglyceride levels [33,36]. After that, hyperglycemia alone does not cause diabetes dyslipidemia or alteration of lipid metabolism [37]. However, IR is the principal determinant factor for diabetes-induced dyslipidemia [37]. Additionally, T2DM brings about dire and long-lasting adverse impacts (Figure 4) on the vascular endothelium among diabetic individuals, causing postprandial glucose (PPG) impales and sustained hyperglycemia. This ends up in the evolution of microvascular (e.g., neuropathy, nephropathy, retinopathy) and macrovascular (e.g., MI, stroke, peripheral vascular disease) complications of the disease [38-42]. Vascular complications start much before the T2DM diagnosis [43]. Consequently, patients frequently suffer from CVDs and their related complications [44]. Hyperglycemia is a sustained and paramount risk feature for the development of diabetic complications, especially CVDs. The reduction of glycated hemoglobin (HbA1c) can decrease the risk of complex disease scenarios. It has been advocated that HbA1c below 6.0% minimizes CVD risk [45].

Lifestyle Interventions and Dietary Factors in the Prevention of CMS and T2DM

The American Heart Association (AHA) recommends addressing three issues to prevent CMS. Those are “eat better, get active, and lose weight” [45]. One study reported, echoing the AHA, that “currently, there is no effective preventive approach beyond lifestyle-based interventions aimed at normalizing body weight and achieving and maintaining cardio-metabolic control, including lipid levels, blood glucose, and blood pressure (BP)” [46].

The amount of food consumption is significantly related to overweight and obesity [34]. Food constituents and characteristics also play a primary role [47-50]. It has been reported that a high intake of refined sugar-containing foods, sweets, sugar-sweetened beverages, deep-fried foods, and red meat contributes to an increased risk of obesity, T2DM, and IR [51,52]. Overall, high consumption of ready-to-eat meat remains the primary perpetrator regarding obesity and T2DM [49,53]. Conversely, those consuming high amounts of fresh fruits, legumes, green leafy vegetables, nuts, wholegrain foods, and fish - especially from the Mediterranean diet - have an antithetical relationship with developing obesity and T2DM [54,55]. Moreover, healthy foods (containing low carbohydrates, fresh fruits, vegetables, etc.) and home-cooked meals protect people from developing obesity, T2DM, and CMS [45,56-59]. Additionally, it has been reported that the development of CMS is significantly associated with eating high-calorie foods, deskbound work, and ghoulish lifestyles, such as obesity and T2DM [60].

Low physical activity and obesity are self-determining predictors of T2DM [61,62]. Obesity increases adipose tissue and is associated with low-grade inflammatory changes [63-65]. Adipose tissue generates several adipokines and free fatty acids (FFAs) [66,67]. Among adipokines, adiponectin is one of the principal components determining obesity, IR, and T2DM [68]. Additionally, raised levels of FFAs, especially saturated FFAs (e.g., stearic and palmitic acid) in plasma, impair insulin secretion and sensitivity and promote glucose intolerance [69-71]. Therefore, several studies concluded that inadequate physical activity raises the possibility of T2DM and CMS [72-75]. T2DM is the dominant cause of untimely loss of life [76,77]. Individuals with T2DM who are poorly therapeutically intervened often give rise to several health complications [78]. T2DM complications include CVDs, stroke, nephropathy, retinopathy leading to blindness, neuropathy, leg and foot ulcers resulting in amputations, and loss of life [79,80].

Weight Loss as a Key Strategy in Managing CMS and T2DM

Healthy lifestyle adoption, especially weight reduction, is the ultimate answer to managing T2DM and CMS [81]. Weight loss possesses the potential to reduce hyperglycemic index; hence, minimizing weight and obesity tends to normalize blood glucose and basal metabolic index (BMI) levels [82,83]. Loss of overweight not only normalizes blood glucose levels but also addresses multiple health problems, such as correcting high blood pressure, hyperlipidemia, and other CVD precipitation features. Concurrently, it reduces the need for antihypertensive, antilipidemic, and antidiabetic medications [84,85]. Loss of weight and normalizing T2DM and its multiple chronic disorders - especially hypertension - promoting vascular health notably diminishes the microvascular and macrovascular obstacles of diabetes [86,87]. All health must be promoted by altering bad food habits, increasing physical activity, and reducing weight [41]. Policymakers must plan to reduce fast food consumption, which contains extra corn syrup, refined sugar, artificial sweeteners, salt, coloring agents, and potentially disease-promoting chemicals such as phosphate preservatives and others [88,89]. Public and private healthcare authorities should develop programs that increase physical activity and amusement [90-92]. Mass media at the community level should be utilized to promote balanced food and highlight the benefits of increased physical activity [93-97]. Health professionals should receive educational intervention to counsel their patients on building healthy eating habits and increasing physical activity to defend themselves from the atrocities of T2DM and CMS [98-103]. Obesity and CMS-related clinical trials published and indexed in PubMed in the last year (https://pubmed.ncbi.nlm.nih.gov/?term=Obesity%2C%20Cardiometabolic%20Syndrome&filter=simsearch2.ffrft&filter=pubt.clinicaltrial&filter=datese, accessed June 30, 2024) are depicted in Table 1 [104-138].

**Table 1:** Depicting clinical trials published last year and indexed in PubMed

Author and journal details	Findings	Conclusion
Freitas and Rodrigues [104]	The treatment group with liraglutide showed a superior effect on reducing abdominal circumference (AC) and trunk fat mass (TFM) compared to the control group treated with sibutramine. An Independent association was found between treatment with liraglutide and TFM.	Obese patients with metabolic syndrome may benefit from treatment with liraglutide as it improves inflammatory and cardiometabolic parameters and lessens TFM correlated with AC.
Shyam et al. [105]	A considerable association was found between increased risk of COVID-19 and higher baseline waist-to-height ratio (WHtR), body mass index (BMI), body weight, and waist circumference.	Significant weight reduction has a beneficial effect on reducing COVID-19 risk in obese/overweight elderly patients.
González-Palacios et al. [106]	Ingestion of ultra-processed food (UPF) was found to be certainly related to fasting blood sugar, HbA1c, BMI, waist circumference, weight, diastolic blood pressure, triglyceride, and triglyceride glucose index.	UPF eating and drinking raises the risk of cardiometabolic syndrome (CMS) in adults.
Konieczna et al. [107]	Patients in the intervention group (energy-reduced Mediterranean diet with escalated physical exercise) displayed higher abatement in total fat and visceral fat and a more remarkable surge of total lean mass compared to the control group. Attainment of improvements needed intervention for at least 12-17 months.	Lifestyle modifications like regular physical exercise and taking a Mediterranean diet with less calorie contributes to raising total lean mass and decreasing visceral and total fat in elderly patients.
Sethi et al. [108]	Inclusion of the ketogenic dietary practice among adult patients with schizophrenia or bipolar disorder who were overweight or had at least one metabolic abnormality showed a substantial decrease in BMI, weight, waist circumference, and visceral fat. It also reduced triglyceride and homeostatic model for insulin resistance.	The ketogenic diet can alleviate cardiometabolic risk, which in turn may increase the general well-being of psychiatric patients.
Deshmukh et al. [109]	More substantial weight loss was observed among morbidly outsized female patients with polycystic ovarian syndrome (PCO) who were prescribed a very low-calorie diet (VLCD) in comparison with patients receiving a moderate energy deficit diet. The first group also reduced free androgen index, waist-to-hip ratio, fasting blood sugar, and sex hormone-binding protein.	The VLCD cohort significantly improved weight reduction, lowering androgen levels, and improving metabolic parameters in PCO patients.
Lauw et al. [110]	This randomized clinical trial among obese/overweight adults found that groups treated with dietary intervention and symbiotic significantly reduced weight loss, insulin, fasting blood sugar, triglyceride, homeostasis model assessment of insulin resistance (HOMA-IR), and a rise in high-density lipoprotein (HDL). This intervention also contributed to lowering body and trunk fat mass and BMI.	Although increased consumption of fruits/vegetables or supplementation of synbiotics may alleviate cardiometabolic risks, combining those two is more competent in treating obesity.
Waddingham et al. [111]	This animal study on lean and obese rats observed that obese rats treated with geranylgeranylacetone (GGA) experienced decreased isovolumic relaxation time of the left ventricle (LV) without any effect on ejection fraction (EF), BP, or weight of LV. GGA also escalated HSPB1 and HSPB5 expression and diminished the stiffness of cardiomyocytes.	Adult patients with CMS who had early diastolic dysfunction could benefit from oral GGA therapy.
Wortelboer et al. [112]	Fecal filtrate transplantation (FFT) from a healthy donor was done on adults suffering from metabolic syndrome, and no statistically significant variance was detected in glucose metabolism between the control and case groups. No severe side effects were noted. However, FFT was associated with significant alteration of the composition of phage virion.	Bacteriophages may contribute to temporary alteration of gut microbiota without serious adverse effects.
Cuatrecasas et al. [113]	Dapagliflozin + metformin in the treatment of T2DM shows a more substantial reduction of weight, waist circumference, omental (om) fat, Om thickness, and right perirenal (RK) fat was noted in comparison to treatment with metformin alone. This combined modality showed efficiency in lessening RK fat.	Dapagliflozin may be considered an effective treatment modality to reduce perirenal fat.
Zhang et al. [114]	Fecal microbial transplantation (FMT) had statistically significantly (p < 0.05) positive effects on metabolic syndrome (MetS) parameters	This study reported that meticulous FMT application reduces MetS.
Feehan et al. [115]	Time-restricted fasting (TRF) was introduced in patients with non-alcoholic fatty liver disease (NAFLD), and more remarkable improvement was seen in reducing liver steatosis, waist circumference, weight, and BMI than the patients who were treated with standard diet and lifestyle advice.	In patients with NAFLD, TRF may be considered an essential tool for reducing cardiometabolic risks, especially weight without implementing calorie restriction.
Garicano et al. [116]	The three months supplementation (S-adenosyl-L-methionine + N-acetylcysteine + thioctic acid + vitamin B6 (MetioNac®)) was given among 27 patients (45-65 years old) metabolic syndrome (MetS) and at-risk of metabolic associated fatty liver disease (MAFLD) on evaluate lipidic and biochemical parameters. The experimental cohort showed statistically significant (p < 0.05) reductions in low-density lipoprotein cholesterol (LDL-C), very-low-density lipoprotein cholesterol (VLDL-C), triglycerides (TG), total cholesterol, and glucose levels. In parallel, raised levels of high-density lipoprotein cholesterol (HDL-C) were observed. Aspartate aminotransferase (AST) and alanine aminotransferase (ALT) levels decreased but were not statistically significant. A reduction in weight was also observed.	The addition of MetioNac® possibly has an effective pharmacodynamic in minimizing IR, hyperlipidemia, obesity, and overweight among MetS cases. This research recommended more studies comprising a larger population.
Laue et al. [117].	One hundred eighty adults who were overweight in the abdominal area were separated into three groups. The first, second, and third cohorts received a placebo, probiotics, and symbiotics, respectively. The probiotic arm showed a more considerable change in body fat mass. The probiotic group also observed positive clinical outcomes in BMI, body weight, waist circumference, visceral fat tissue, liver steatosis, and waist-to-height ratio. In the symbiotic arm, improvements were seen in liver steatosis and visceral fat tissue.	Overweight and its parameters possibly improved using a probiotic mixture and symbiotic.
Nilsen et al. [118]	Obese adults with or without metabolic syndromes (MetS) underwent a lifestyle modification course. Bleeding on probing and stage III/IV periodontitis were found to be more in the non-MetS wing. The significant impact of age was observed in the case of stage III/IV periodontitis when adjusted for MetS and variables related to obesity.	In obese persons, the development of periodontitis does not depend upon the presence of MetS. In severe periodontitis (III/IV), age significantly impacts MetS and obesity-related variables.
Moreira et al. [119]	Severely obese women with or without metabolic syndrome who underwent Roux-en-Y gastric bypass (RYGB) surgery displayed significant positive changes in body mass index (BMI), body weight, glucose levels, low-density lipoprotein (LDL) cholesterol, total cholesterol, high-density lipoprotein (HDL) cholesterol, leptin, adiponectin, and the ratio between adiponectin and leptin.	RYGB can be an option for severely obese women to induce weight loss and gain progress in systemic and biochemical parameters.
Bouzas et al. [120]	Elderly obese adults with MetS were assessed for diet and dietary patterns, monetary overhead of the diet, health variables, and severity of MetS. The diet cost was bluntly connected with higher energy expenditure, education, non-smoking, higher blood glucose, and truncal obesity. It was inversely related to higher triglyceride (TG) and decreased high-density lipoprotein cholesterol (HDL-C) prevalence. Healthy dietary patterns are usually expensive and contain less energy.	A cheaper diet was associated with a higher incidence of cardiometabolic risks. A healthier diet costs more money, which may act as an obstacle to preventing chronic health problems related to diet.
Candás-Estébanez et al. [121]	Two hundred-two patients with MetS were divided into two groups. The intervention and control group received an energy-restricted Mediterranean diet (er-MedDet) plus physical exercise and an er-MedDet only, respectively. The intervention group experienced significant weight loss, reduction in BMI, waist circumference, TG, LDL, and non-HDL cholesterol, and improved HDL-C. Advanced lipoprotein testing (ADLT) was conducted. It detected a reduction in small-dense-LDL-C, intermediate-density lipoprotein, VLDL-TG, and HDL-TG and an increment of Large LDL and VLDL particles among the intervention cohort.	Combining physical exercise and an er-MedDet alleviated cardiovascular risks in MetS.
Gioxari et al. [122]	Patients with truncal obesity and MetS receiving chios mestiza oil displayed considerable positive clinical changes in TG, LDL, systolic BP, alanine aminotransferase, weight, body and visceral fat, oxidized LDL, and adiponectin. Overall quality of life was also observed in the intervention group.	Chios mestiza oil possess efficient and prudent pharmacological potential. Thus, it can serve as an adjunct therapy for obesity and MetS.
Misella et al. [123]	Ingestion of the New Nordic Renal Diet (NNRD) by chronic kidney disease (CKD) patients showed a more considerable reduction of systolic BP, abdominal fat, body weight, and improved renal parameters.	NNRD may reduce cardiometabolic risk factors in patients with moderate CKD.
Vinding et al. [124]	In a long-term follow-up, children whose mothers took fish oil supplementation during the gestational period showed an increased risk of higher BMI, overweight, and raised body and fat mass. Their metabolic score was also higher than that of the control group.	Fish oil supplementation during pregnancy may result in higher BMI, increased risk of becoming overweight and developing fat mass, and more healthy cardiometabolic score.
Rossello et al. [125]	An intensive lifestyle intervention (ILI) comprising energy-restricted MedDet, regular aerobic physical workouts, and cognitive-behavioral weight management was prescribed to elderly overweight patients. Although weight reduction was more prominent in the intervention wing, left atrial (LA) structure and functions deteriorated in both groups.	ILI is not efficacious in obese or overweight adults with metabolic syndromes.
Gawlik-Kotelnicka et al. [126]	Adult patients with depressive disorder and MetS were recruited, and the intervention groups received several strains of probiotics and excipients, whereas the placebo group took only excipients. The probiotic wing showed bottom-level clinical improvement; nevertheless, it is statistically significant. Psychomotor parameters in self-assessment were less improved, whereas metabolic abnormalities were more.	Patients with depression may use probiotics as an adjunct therapy, but the presence of liver steatosis and obesity may influence the outcome. However, more extensive scaled studies are required.
Samarasinghe et al. [127]	Eighty obese adult women with polycystic ovarian syndrome (PCOS) were divided into two groups. The intervention group has undergone vertical sleeve gastrectomy, and the control group received behavioral therapy and medical treatment. Significant spontaneous ovulation was observed in the intervention group, although short-term complications were noted more than in the medical group.	The success rate of induced spontaneous ovulation is higher with bariatric surgery in obese adult females with PCOS.
Zeng et al. [128]	Decaffeinated green tea extract (GTE) administration into adult patients with or without metabolic syndrome reduced serum exotoxin, increased circulating catechins and valerolactone levels and decreased fasting plasma glucose.	Catechin-rich GTE efficiently reduced endotoxin and improved glucose control in adults with or without metabolic syndrome.
Garcia-Beltran et al. [129]	Non-obese adolescent girls with PCOS were divided into two groups. One group was treated with oral contraceptives (OC), and the other one with spirit. Girls without PCOS were considered as controls. SPIOMET (SPI (spironolactone)-PIO (pioglitazone)-MET (metformin)) groups did not show any change in ALT and gamma-glutamyl transferase (GGT) levels. Still, the OC group observed raised levels during the treatment phase only. Lower diazepam-binding protein-1 (DBI) levels were noted in the OC group, and raised fibroblast growth factor-21 (FGF21) levels were found in girls with PCOS. Still, meteorin-like protein (METRNL) showed no difference between the control and experimental groups. In the OC-treated wing, ALT and GGT levels were directly correlated with METRNL levels in blood.	Treatment with OC raises METRNL levels by enhancing GGT and ALT concentrations in adolescent girls with PCOS.
Citarrella et al. [130]	Patients with metabolic syndrome and glucose intolerance or T2DM were prescribed a unique food supplement comprising D-chiro-inositol, glucomannan, *Cinnamomum zeylanicum Blume*, and inulin. At 16 weeks of follow-up, it was observed that body weight, BMI, insulin, and Homa index were reduced, and there was a more remarkable improvement in TG, cholesterol, and LDL levels.	This unique food supplementation is a safe and effective approach to decreasing MetS and its associated risk factors.
Pandey et al. [131]	Obese or overweight adults without DM were given subcutaneous injections of liraglutide, and after forty weeks, thigh muscle fat and adverse muscle composition were decreased in the intervention group.	Injectable liraglutide pharmacology contributes to reducing muscle fat, but further studies are needed.
Wang et al. [132]	Women with PCOS and obesity underwent lifestyle modification, including calorie-restricted diet and physical exercise. After completion of the intervention, they were divided on their ovulation status into resumed ovulation (OR-) and anovulatory group. Only AMH concentration was significantly lower in the OR- group. Anthropometric measures, baseline characteristics, insulin, FAI, T, and HOMA-IR did not vary significantly.	Women with PCOS and obesity may benefit from androgen level monitoring after lifestyle intervention, although further studies are needed.
ELM Trial Research Group [133]	Patients suffering from MetS were recruited from five various geographic areas and were prescribed a lifestyle modification intervention and subsequently followed up. Truncal obesity and higher BP or BP-lowering agents were found to be the most expected metabolic elements.	Rigorous lifestyle alterations, especially healthy dietary practices with increased aerobic physical activity, effectively abrogate MetS. Hence, it reduces the growing number of MetS-related public issues.
Finkelstein et al. [134]	Participants with metabolic syndrome were given dietary advice along with physical exercise. A predicted BMI score corresponding to fat-related clinical measurement was measured using an MRI of the brain. Reduced BMI prediction score was noted to be associated with actual body weight loss and was significantly greater in the intervention wing than in the control wing.	As a neural biomarker, predicted BMI is unique for reduced weight and obesity-related neuronal modifications.
Suder et al. [135]	Adult male persons with metabolic syndrome who underwent aerobic exercise showed a reduction in asprosin (ASP) level, whereas men not doing exercises experienced a rise in ASP level. Participants who practiced aerobic and resistance training did not develop reduced ASP but an increased fat-free mass/fat mass ratio and decreased insulin level.	Both aerobic resistance and aerobic exercise may contribute to decreasing visceral fat tissue.
van Brakel et al. [136]	Plant stanol-added products were consumed by overweight/obese persons at least two weeks before receiving the COVID-19 vaccine and continued up to four weeks. The intervention group developed an increased IgM titer than the control group. Spike concentration of IgG was also found to be elevated in the intervention group, with a decrease in cytokine level.	Consumption of plant stanols enhances immunity against COVID-19 in adults with metabolic syndrome, which may benefit the patients at risk of COVID-19 complications.
Ismail and Hamed [137]	Significant improvements in BMI, psoriasis severity, erectile dysfunction (EF), BP, abdominal circumference, HDL, TG, and fasting blood sugar were observed in obese patients with psoriasis, EF, and metabolic syndrome after the change of lifestyle comprising of a low-calorie diet and treadmill walking.	Significant weight change can be achieved after lifestyle modification in obese patients with psoriasis, which may alleviate psoriasis severity and its associated complications.
Chun et al. [138]	A multicomponent care model of communication and information technology, biogenetic markers, and patient-reported outcome measures were developed. Its effects were evaluated in T2DM young adult patients. The intervention group showed lower HbA1c, less incidence of hypoglycemia, higher BP, and use of antihypertensives.	This multicomponent intervention program may help young diabetic patients to accomplish specific metabolic targets.

Psychological and Behavioral Interventions in Managing CMS and T2DM

The biopsychosocial model of health emphasizes the critical role of behavioral and social factors in managing CMS and T2DM, and the beneficial effects of integrating behavioral and biomedical interventions on health outcomes. Psychological factors, such as depression, diet-related psychological characteristics, and sleep quality, may have a significant impact on the progression of diseases and the effectiveness of prevention and therapeutic interventions [139]. Promoting lifestyle changes aimed at weight loss, such as instilling healthy dietary habits and encouraging increased physical activity, can be achieved through evidence-based behavioral interventions. For example, cognitive-behavioral therapy (CBT), which involves changing cognitive and behavioral patterns, has shown effective blood glucose management among diabetic patients and has generally demonstrated positive outcomes [140]. Motivation interviewing, an approach that emphasizes self-management, and places the responsibility for the treatment outcomes on a patient, showed an overall positive effect on emotional distress, depression, and self-efficacy [141].

Another important aspect contributing to improved treatment outcomes is providing psychosocial support and empowering patients with chronic diseases, such as CMS and T2DM. It has been shown that upon implementing psychosocial support programs patients' adherence to treatment and overall quality of life significantly improved. A recent study has indicated that elderly diabetic patients with a positive social environment are less likely to experience depression and benefit from opportunities to engage and maintain their life purpose [142]. On top of fostering a supportive social environment, patient education and empowerment initiatives play pivotal roles in the effectiveness of preventative and therapeutic interventions. Strategies to improve health literacy, enhance lifestyle habits, and develop self-management skills should be tailored to individual patients, considering family dynamics and beliefs [143,144].

Limitations of the study

This paper provides an overview of the global impact of T2DM, CMS, and cardiovascular complications, the complex dynamics of their interrelationship, and ways to mitigate the effects of these medical conditions through lifestyle changes and dietary modifications. However, this study has several limitations. Firstly, only published studies were analyzed, which might have affected the representativeness of the sources, as unpublished findings were omitted. Secondly, as with most review papers, our study may be prone to publication bias due to the higher chances of studies demonstrating significant findings being published [145]. Additionally, as NCD research is a fast-evolving field, newly emerging evidence may alter the views shared in this paper. Lastly, although strategies for preventing and controlling CMS and T2DM were outlined, practical ways of their implementation in different contexts, including specific barriers and the ways to address them, were beyond the scope of this paper.

Future recommendation 

The findings from this narrative review paper highlight the importance of enhanced training among healthcare providers on different aspects of the prevention and treatment of CMS, as well as associated T2DM and CVDs. Healthcare workers should be encouraged to familiarize themselves with new research findings related to CMS, enabling evidence-based interventions to improve patient outcomes [81,146].

**Figure 5:**
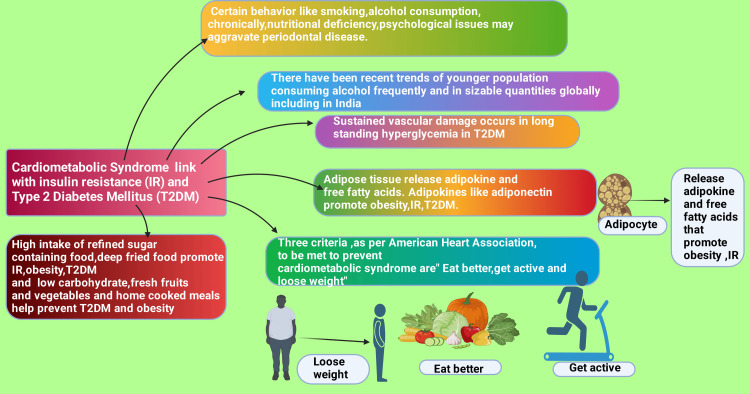
Diagram depicting the graphical abstract Note: This figure has been drawn using the premium version of BioRender [11] (https://biorender.com, accessed on July 18, 2024) with the agreement license number KF272Q992M. Image credit: Rahnuma Ahmad

Focusing on the following key aspects would be beneficial from a research perspective. Firstly, there is a need to prioritize biomedical and clinical research to examine the processes underlying the development and progression of CMS and T2DM. Prospective longitudinal studies will provide invaluable insights into CMS causality and long-term effects. These studies will help identify the factors contributing to the progression of medical conditions and assess the effectiveness of novel preventative and therapeutic interventions. The studies should be conducted in different settings and populations, such as low-income urban or rural dwellers, marginalized or outreach communities, to ensure that the equity of healthcare provision is addressed and that the social gradient in health is reduced. Finally, it would be valuable to apply a multidisciplinary approach in future research on CMS, including behavioral scientists, nutritionists, and other health professionals alongside biomedical and clinical researchers [147]. The graphical abstract of this narrative review is illustrated in Figure 5.

## Conclusions

This paper explores the complex interconnections between CMS, T2DM, and CVDs. IR serves as the main trigger for detrimental physiological changes in diabetic patients, with two main implications being hyperglycemia and dyslipidemia. These, in turn, initiate a cascade of adverse macro- and microvascular effects, including hypertension, stroke, PAD, CAD, neuropathy, nephropathy, and retinopathy. The risk of complex disease scenarios can be significantly reduced by maintaining HbA1c levels within the normal range.

Long-term prevention strategies for CMS and T2DM are based on lifestyle changes, such as dietary modifications aimed at increasing the intake of fruits, vegetables, and whole grains and encouraging higher physical activity levels. Maintaining a healthy body weight is one of the key strategies in preventing and controlling metabolic diseases. Lifestyle-based interventions to address metabolic diseases are grounded in the biopsychosocial model of health, which implies an integrative approach combining biomedical and behavioral interventions. Some behavioral interventions, such as CBT, motivational interviewing, and supportive social environments, have demonstrated promising results. These contribute to the body of evidence on the effectiveness of the patient-centered model for achieving positive lifestyle changes, especially among elderly patients.
